# L-carnitine and PPARα-agonist fenofibrate are involved in the regulation of Carnitine Acetyltransferase (CrAT) mRNA levels in murine liver cells

**DOI:** 10.1186/1471-2164-15-514

**Published:** 2014-06-24

**Authors:** Klemens Kienesberger, Aniko Ginta Pordes, Thomas Georg Völk, Reinhold Hofbauer

**Affiliations:** Centre for Molecular Biology, Department of Biochemistry and Cell Biology, Max F. Perutz Laboratories, University of Vienna, Dr. Bohrg. 9, Vienna, A-1030 Austria; Department of Medical Biochemistry, Division Molecular Genetics, Max F. Perutz Laboratories, Medical University of Vienna, Dr. Bohrg. 9, Vienna Biocenter, A-1030 Vienna, Austria; Baxter Innovations GmbH, A-Wagramer Str. 17-19, Vienna, 1221 Austria

**Keywords:** L-carnitine, PPARα, Carnitine acetyltransferase, Fenofibrate

## Abstract

**Background:**

The carnitine acetyltransferase (CrAT) is a mitochondrial matrix protein that directly influences intramitochondrial acetyl-CoA pools. Murine CrAT is encoded by a single gene located in the opposite orientation head to head to the PPP2R4 gene, sharing a very condensed bi-directional promoter. Since decreased CrAT expression is correlated with metabolic inflexibility and subsequent pathological consequences, our aim was to reveal and define possible activators of CrAT transcription in the normal embryonic murine liver cell line BNL CL. 2 and via which nuclear factors based on key metabolites mainly regulate hepatic expression of CrAT. Here we describe a functional characterization of the CrAT promoter region under conditions of L-carnitine deficiency and supplementation as well as fenofibrate induction in cell culture cells.

**Results:**

The murine CrAT promoter displays some characteristics of a housekeeping gene: it lacks a TATA-box, is very GC-rich and harbors two Sp1 binding sites. Analysis of the promoter activity of CrAT by luciferase assays uncovered a L-carnitine sensitive region within −342 bp of the transcription start. Electrophoretic mobility shift and supershift assays proved the sequence element (−228/-222) to be an L-carnitine sensitive RXRα binding site, which also showed sensitivity to application of anti-PPARα and anti-PPARbp antibodies. In addition we analysed this specific RXRα/PPARα site by Southwestern Blotting technique and could pin down three protein factors binding to this promoter element. By qPCR we could quantify the nutrigenomic effect of L-carnitine itself and fenofibrate.

**Conclusions:**

Our results indicate a cooperative interplay of L-carnitine and PPARα in transcriptional regulation of murine CrAT, which is of nutrigenomical relevance. We created experimental proof that the muCrAT gene clearly is a PPARα target. Both L-carnitine and fenofibrate are inducers of CrAT transcripts, but the important hyperlipidemic drug fenofibrate being a more potent one, as a consequence of its pharmacological interaction.

**Electronic supplementary material:**

The online version of this article (doi:10.1186/1471-2164-15-514) contains supplementary material, which is available to authorized users.

## Background

L-carnitine (L-3-hydroxy-4-N – trimethylaminobutyrate) is a low molecular ammonium compound, which is synthesized from the two essential amino acids lysine and methionine mostly in liver and kidney [1–3]. It is an important cofactor for the transport of long chain fatty acids across the mitochondrial membranes into the matrix where they can be broken down by ß-oxidation to acetyl-CoA to obtain energy via the citric cycle [4, 5]. Due to its regulative functions on the acetyl-CoA pools and its transporting features of acyl groups, L-carnitine covers also a key role in glucose metabolism and assists in fuel sensing [6]. An abnormal increase of intramitochondrial acetyl-CoA concentration in liver cells leads to increased gluconeogenesis, one aspect of diabetes [7]. High levels of acetyl-CoA have also been associated with abnormalities in skeletal muscles of diabetic patients, where insulin seems to be unable to mediate the switch from lipid to glucose metabolism leading to decreased glucose utilization [8].

Carnitine Acetyltransferase (CrAT) is a mitochondrial matrix enzyme, which transfers short acyl groups from acyl-CoA to L-carnitine resulting in an acetyl-carnitine-ester [9]. Therefore it defines the equilibrium of acetyl-CoA (+free L-carnitine) and acetylcarnitine (+free CoA) [10]. Highest expression levels of CrAT are reached in muscle cells and testis [9]. Although expression levels are low in liver it exerts a significant metabolic function by regulating acetyl-CoA pools at the crossroads of anabolic and catabolic pathways [11]. Carnitine supplementation promotes CrAT-mediated acetylcarnitine efflux and improves metabolic outcome in obese rodents [12, 13]. Furthermore the importance of CrAT has been recently underlined by the fact that muscle-specific knock-out mice showed increased metabolic inflexibility, meaning that they failed to adjust appropriately to mitochondrial fuel selection in response to nutritional cues [14]. Another very supportive fact for our study was that NIDDM patients showed decreased levels of CrAT mRNA levels [14]. These findings render CrAT to be an interesting pharmacological target for treatment of NIDDM. L-carnitine itself could be a pharmacological tool since supplementation after artificially induced L-carnitine deficiency induces CrAT expression in a moderate way [15].

Murine *CrAT* gene (GeneID 12908) had been mapped on chromosome 2 next to the protein phosphatase 2A, regulatory subunit B (PR 53) gene (*PPP2R4*). No detailed promoter studies have been published so far, but PPARα plays a key role in CrAT transcription control [16, 17]. So we hypothesized that a cooperative interplay between L-carnitine and PPARα positively influences CrAT expression.

## Results

### L-carnitine and PPARα induce murine CrAT expression

The L-carnitine levels in the FCS after dialysis against phosphate buffered saline for 48 h dropped significantly from 36 μM to 16 μM (see Table 1). In a direct consequence the intracellular L-carnitine levels after cultivation in dialyzed FCS were reduced more than 70% and after L-carnitine supplementation could be restored to more than 70% of the original level of 973 μM L-carnitine (see Table 2). Under these cell culture conditions we performed our L-carnitine deprivation/supplementation studies.

**Figure 1:**
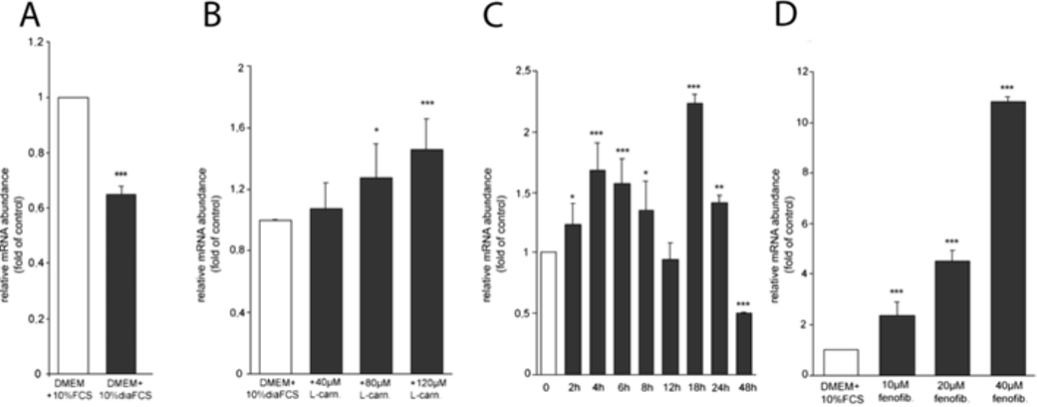
**Quantification of CrAT mRNA levels. (A)** TIB-73 murine liver cells were cultivated in DMEM and 10%FCS in comparison to cells treated 24 h with dialyzed FCS. **(B)** TIB-73 murine liver cells were grown for 24 h with dialyzed FCS and supplemented afterwards for 4 hours with L-carnitine (40–120 μM). **(C)** Following 24 hours of treatment with dialyzed FCS TIB-73 cells were supplemented with 80 μM L-carnitine for 2–48 h. A second L-carnitine boost (80 μM) took place after 15 hours. Mean value for 0 h L-carnitine suplementation was designated as 1. Supplemented cultures were compared to non-supplemented control (DMEM + 10%diaFCS) in B and C. Values represent means ± SD (n = 3). Means without asterisk show no statistical significance (p > 0.05); (p-values of asterisk marked means are as followed: *p < 0.05, **p < 0.01, ***p < 0.001) **(D)** TIB-73 cells were grown in DMEM + 10%FCS for 24 hours and afterwards treated with fenofibrate (10–40 μM). Values represent means ± SD (n = 3). Supplemented cultures were compared to physiological control. ***p < 0.001.

Our first experiments examined the regulation of the murine CrAT gene by L-carnitine and fenofibrate. CrAT mRNA levels were increased by L-carnitine supplementation (for 4 h) after artificially induced L-carnitine deficiency (Figure 1A). Additionally we tested the influence of 80 μM L-carnitine on muCrAT mRNA levels up to 48 hours after supplementation. After 4 hours the first peak of CrAT mRNA levels was detected (1.65 fold increase). A second application rate of 80 μM L-carnitine was added after 15 h and lead to even higher CrAT mRNA levels. (2.23 fold after 18 h) (Figure 1B). Also the PPARα-agonist fenofibrate induced muCrAT up to 11-fold (with 40 μM fenofibrate) (Figure 1C).

**Table 1:** **Serum L-carnitine Levels**

	Serum L-carnitine concentration (μM)	SD	p-value
(A) Fetal Calf Serum	36	±9	-
(B) Dialysed Fetal Calf Serum	16	±5	p = 0.028

Measurements of serum L-carnitine concentrations before and after dialysis. For statistical analysis dialysed FCS was compared to non-dialysed FCS (values represent means, n = 3, (A) vs. (B) p = 0.028).

**Table 2:** **Intracellular L-carnitine Levels**

	Intracellular L-carnitine concentration (μM)	SD	p-value
(A) TIB73 in DMEM + 10%FCS	973	±52	-
(B) TIB73 in DMEM + dia.FCS	265	±51	p < 0.001
(C)TIB73 in DMEM + dia.FCS +80 μM L-carnitine	711	±72	p < 0.001

Measurements of intracellular L-carnitine concentrations of TIB73 cells under physiological conditions (A), L-carnitine deficiency (B) and supplementation (C). For statistical analysis conditions A and B were compared as well as conditions B and C.

(Values represent means, n = 3; (A) vs. (B) p < 0.001 and (B) vs. (C) p < 0.001).

### L-carnitine raises PPARα presence in the nucleus

**Figure 2:**
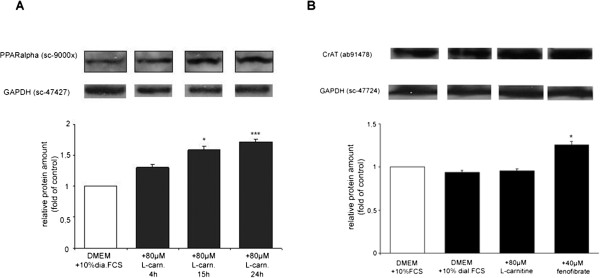
**PPARα Western blot from nuclear extracts of TIB-73 cells. (A)** Cells were treated as described in Figure 1. Nuclear extracts were prepared after 4, 15 and 24 hours of L-carnitine supplementation; values are mean ± SD, n = 3, *p < 0.05 and ***p < 0.001 vs. DMEM + 10% dia.FCS **(B)** CrAT Western blot from whole cell extracts of TIB-73 cells. Cells were treated as described in Figure 1. values are mean ± SD, n = 3, *p < 0.05 vs. DMEM + 10 % dial.FCS.

PPARα protein levels in the nucleus increase constantly after L-carnitine supplementation (Figure 2A). Already 4 hours after L-carnitine supplementation slightly increased levels of PPARα levels are detectable in the nucleus (1.2 fold) and gets more distinct after longer supplementation periods (1.5 fold after 15 hours and 1.7 fold after 24 hours. The western blot presented in Figure 2B shows the almost even induction pattern of CrAT protein levels in TIB-73 cells cultivated in fetal calf serum, dialyzed fetal calf serum and after L-carnitine (80 μM) and/or fenofibrate (40 μM) supplementation. The cultivation in dialyzed fetal calf serum slightly reduces the TIB73 CrAT protein levels and subsequent L-carnitine does not really influence the steady state CrAT protein. Only fenofibrate is able to increase it distinctly.

### L-carnitine increases CrAT promoter activity

**Figure 3:**
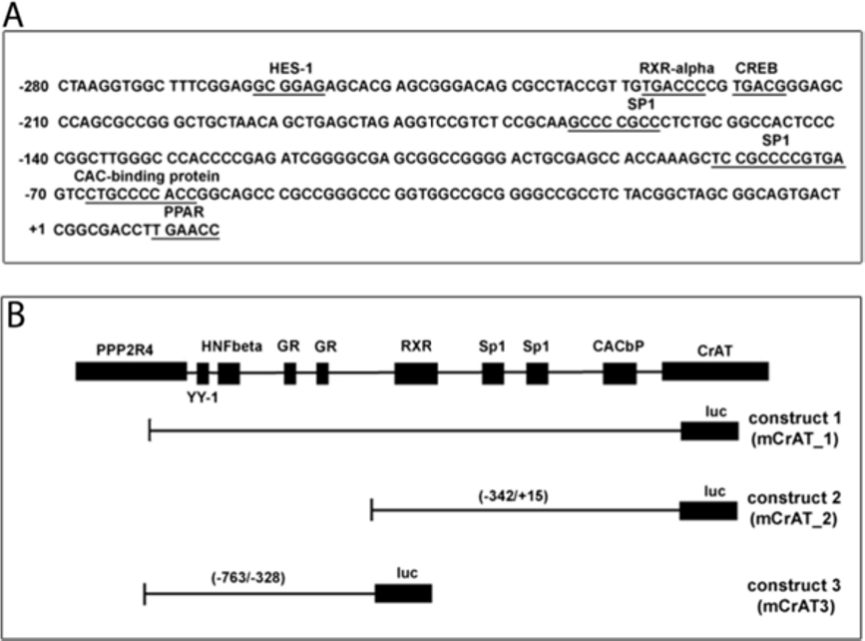
**Organisation of the murine CrAT promoter. (A)** Presentation of the 5′ flanking sequence of the murine CrAT gene. Consensus binding sites are underlined. **(B)** Schematic structure of the murine CrAT promoter with putative binding sites. mCrAT-1 represents the construct for luciferase-assays ranging from −342 bp to +15 bp and mCrAT-2 from −763 to −328 bp. The artificial promoter construct mCRAT-1 contained the RXR-box, two Sp1 elements and one CACbP region in front of the luc-gene of the pGL2-basic luciferase reporter vector. The promoter construct mCRAT-2 contained three YY-1 sites, two GR1 elements and a HNFβ box in front of the reporter luc-gene.

**Figure 4:**
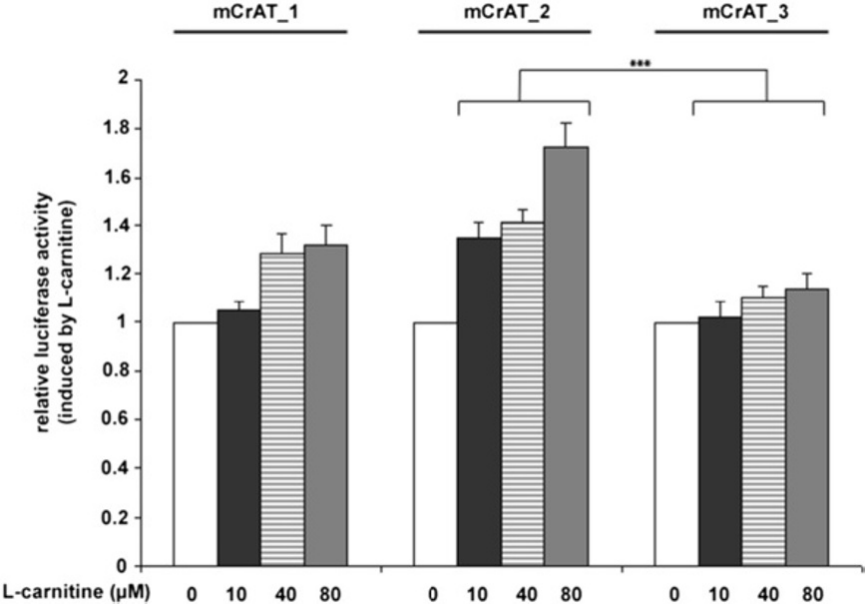
**Activity of different CrAT promoter constructs after supplementation with L-carnitine.** TIB-73 cells cultured in medium containing 10% FCS to cause artificial L-carnitine deficiency were cotransfected either with mCrAT-1, mCrAT-2 or mCrAT-3 together with pCMV-ßgal and supplemented with increasing concentrations of L-carnitine for 4 hours. Luciferase activity was normalized for ß-gal activity. Data represent the mean ± SD, n = 4; the mean value for non-supplemented cultures is designated as 1. Comparison of mCrAT-2 and mCrAT-3 constructs at all 3 supplementation levels for 10, 40 and 80 μM L-Carnitine revealed ***p-values (p < 0.001) indicated with parentheses. A Kruskal-Wallis test for all three constructs resulted in slightly less significant p-values (p = 0.094 for 10 μM L-carnitine, p = 0.0002 for 40 and 80 μM L-carnitine).

Three luciferase constructs (mCrAT-1, mCrAT-2 and mCrAT-3) were designed containing different regions of the 5′flanking region of the murine CrAT gene (Figure 3). All three constructs were transfected separately into TIB-73 murine liver cells and promoter activity was measured after 4 h (Figure 4). mCrAT-2 shows higher promoter activity than mCrAT-3 at all 3 different L-carnitine supplementation levels. Within this construct a RXRα, two Sp1, a CAC-binding protein and a PPARα binding site were identified using the Transfac database via the online-tools TESS and PATCH.

### Analysis of the murine CrAT 5′ flanking sequence

The murine CrAT promoter and its putative cis-regulatory elements for nuclear factors are depicted in Figure 3. Analysis of the 5′-flanking region of exon 1 revealed several putative transcription factor-binding sites. Most importantly a RXRα element was found at −228 bp to −222 bp. The CrAT promoter is a TATA-less one with two SP1 binding sites. Further on the 5′ promoter region harbors binding sites for HES-1, CREB, CAC-binding protein and at last a PPARα site within the first exon.

### L-carnitine raises binding affinity of nuclear extracts to RXRα probe – anti-PPARα antibody abrogates this DNA-protein formation

**Figure 5:**
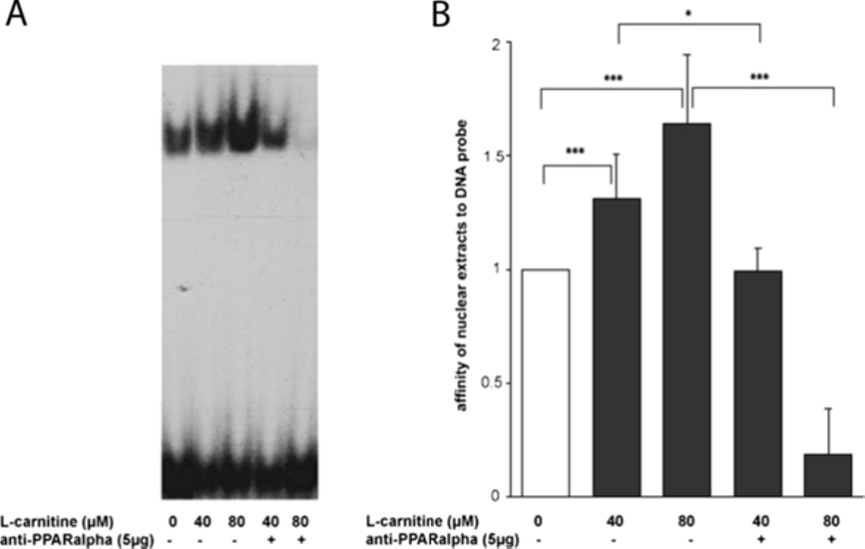
**Protein complexes binding to RXRα binding site at the CrAT promoter. (A)** EMSA. Nuclear extracts from TIB-73 cells supplemented with increasing concentrations of L-carnitine were incubated with γ-^32^P-labeled oligonucleotides representing the RXRα-binding site with anti-PPARα as indicated. **(B)** Histogramm of the denstitometrical scan of the EMSA presented in A. Values are mean ± SD, n = 3, ***p < 0.001 and *p < 0.05 vs. Corresponding supplementation levels are indicated with parentheses and asterisks on top to indicate the statistical significance of the p-values.

To gain more insight into the binding affinities of nuclear extracts to different probes representing the putative transcription factor binding sites we performed several EMSAs. Nuclear extracts of TIB-73 cells were prepared after 4 h of L-carnitine supplementation at different concentrations. EMSA analysis of the DNA probe representing the RXRα binding site revealed increased affinity of nuclear extracts to this ODN with rising L-carnitine concentrations. Additional administration of anti-PPARα antibody (sc-9000X) directly to the EMSA reaction mix lead to weakening of the band shift signal at 40 μM L-carnitine and nearly to extinction of the signal at 80 μM L-carnitine (Figure 5A, B). Based on this result we concluded that L-carnitine influences transcriptional activation via PPARα.

**Figure 6:**
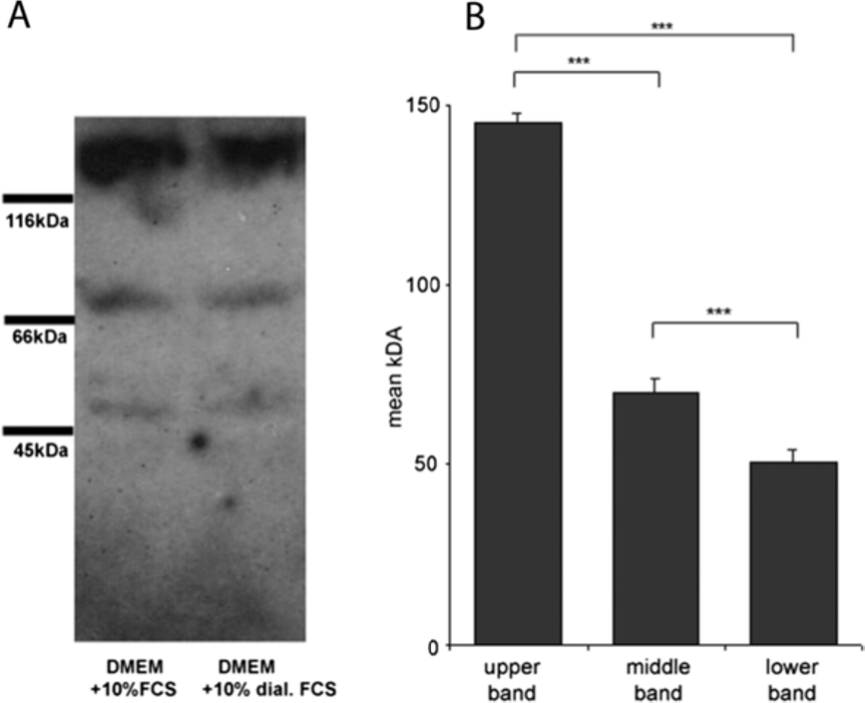
**South-Western Blot of nuclear extracts from TIB-73. (A)** cells cultivated in DMEM + 10 % FCS and DMEM + 10 % dialyzed FCS. Marker proteins adjacent to the blot indicate the size range 116 kD, 66 kD and 45 kD. **(B)** Graphical analysis of 3 distinct bands in kDa (145 ± 3, 70 ± 4, 51 ± 4), values are mean ± SD, n = 4. ***p < 0.001.

Further on we performed Southwestern Blotting to gain more information about the complexity of the protein factors binding to this specific RXRα promoter element. We received three distinct signals at 51 kDa, 70 kDa and 145 kDa. A search in the Transfac database via *molwsearch* (http://www.gene-regulation.com) presented LXRα and PPARα as candidate factors for the 51 kDa signal, c-Myb and cMyc as putative factors for the 70 kDa band and Evi-1 for the 145 kDa signal (Figure 6A, B).

## Discussion

No detailed promoter study of the murine CrAT promoter has been published so far. Basically the CrAT gene exhibits the typical characteristics of a housekeeping promoter: it harbours no TATA box, is GC rich and has two Sp1 binding sites. The distance to the transcriptional start of the opposing PPP2R4, which is encoded on the complementary strand, is only 586 bp. This leads to the reasonable postulation that this promoter very likely is a bi-directional one. For the human PPP2R4 promoter it could be shown that Yin-yang 1 (YY-1) is essential for core promoter activity and that it is a p53 target gene [18, 19]. By applying TESS we could find three YY-elements, two at positions −52 to −44 and +27 to +35 relative to the PPP2R4 transcription start and a third one already in the first exon of the PPP2R4 gene. These putative YY-binding sites very likely represent the murine equivalents to the human promoter (Figure 3).

One aim of our work was to identify inducers of transcriptional activation of CrAT. As depicted in Figure 1 L-carnitine and fenofibrate are such transcriptional activators. L-carnitine induces CrAT and other members of the “acylcarnitine shuttle system” like CPT1a and b, as well as CPT2 transcription levels in the human system similar to mice [15]. Beyond that, in a parallel chip-screen study performed by our lab, we observed that hundred of genes throughout the whole genome are transcriptionally in- or decreased by L-carnitine, underlining the importance of this metabolite [20]. In case of the murine CrAT we observed a rather moderate increase of mRNA levels (up to 1.8 fold) after 4 h of L-carnitine supplementation following artificially induced L-carnitine deficiency. The above-mentioned opposing PPP2R4 gene is transcriptionally induced by L-carnitine and fenofibrate very similar to the CrAT gene as shown in the human liver cell line Hep G2 (see Additional file 1: Figure S1), which is another argument for the bi-directionality of the promoter.

The important hyperlipidemic drug fenofibrate is a much more potent inducer of CrAT transcription levels (up to 11-fold after 3 hours of fenofibrate treatment). But no firm indications exist for a PPRE element in CrAT promoter from bioinformatical analysis. This was also assumed for CPT1a and CPT2 [10], but for the latter PPREs could finally be defined [21]. CPT2 hosts a special PPRE, namely only one half proportion with perfect consensus sequence (TGACCT) [22]. Our results undoubtedly prove that muCrAT is a PPARα target, as it has also been indicated in experiments with PPARα knock-out mice [16, 17].

By reportergene assays we were able to define an L-carnitine sensitive region within 342 nt upstream the transcription start (Figure 3B and 4). Within this sequence many different putative binding sites for nuclear factors were predicted in silico. Our band shift experiments clearly revealed one RXRα element to be sensitive to L-carnitine supplementation.

Based on our data we propose the sequence *TGACCC*CG*TGACGG* at −238 upstream the transcription start to be a possible L-carnitine sensitive RXRα/PPARα binding site. Band shift assays performed with an oligo desoxynucleotide carrying only the 5′-half of this sequence element showed sensitivity to L-carnitine supplementation. At least the sequence box TGACC is present in the CrAT L-carnitine binding site, therefore we propose in analogy to the CPT2 gene this sequence element being a further imperfect but functional PPRE site. Increasing L-carnitine levels lead to enhanced binding affinity of nuclear extracts. Interestingly the application of anti-PPARα to the band shift reaction mix lead to a mitigation of the band shift signal. With increasing L-carnitine supplementation levels the band shift signal almost disappeared, indicating the interference of anti-PPARα with the DNA-protein complex. Such fading out effects have been observed before and were accepted as an experimental proof for a supershift [23]. To rule out that this explicit attenuation effect was an unspecific one, caused by the use of antibodies in general, Supershift assays were performed with antibodies against several candidates of nuclear receptors (see Additional file 1: Figure S2). These validation experiments did not result in any significant changes of the DNA-protein formation except for PPARα. Therefore this effect had to be qualified as a specific interaction.

In addition to this effect we were able to reveal an additional close connection between the PPAR-system and L-carnitine. Transcription levels of PPAR-binding protein, PPARbp, are also inducible by L-carnitine. Chip screen experiments as well as quantitative PCR also provided experimental proof for this effect [20]. Li JL et al. found experimental proof for an interaction of PPARα and L-carnitine as a protective response to oxidative stress [24]. This finding provides additional support to our observations that L-carnitine shows cooperativeness with the PPAR-system. This supposition is strengthened by the results of the Western blot depicted in Figure 2A: L-carnitine supplementation leads to increased levels of PPARα in the nucleus. Obviously increased L-carnitine levels foster the translocation of PPARα into the nucleus. It is of fundamental nutrigenomic importance that RXRα/PPARα heterodimers are positively regulating transcription rates of fatty acid degradation genes as counterparts to LXRα and SREBP1-c, factors which mostly induce anabolic acting fatty acid synthesis genes [25, 26].

Carnitine Acetyltransferase is a central regulator of intramitochondrial acetyl-CoA pools. The latter holds a prominent position in intermediary metabolism as the universal end product of fatty acid, glucose and amino acid oxidation [12, 14]. Acetyl-CoA excess leads to blockage of the TCA cycle and subsequently to accumulation of short carbon compounds, which are further on used for gluconeogenesis in liver [7, 27]. This is besides insulin resistance another factor of the pathogenesis of NIDDM [28].

Recently the pivotal role of CrAT in acetyl-CoA metabolism was confirmed since experiments with CrAT^M−/−^ knock-out mice showed that absence of CrAT leads to accumulation of medium and long chain acylcarnitines in muscles and subsequently to glucose intolerance via overloaded mitochondria [14]. These findings indicate a possible positive effect of L-carnitine and fenofibrate in the regulation of glucose homeostasis via direct transcriptional activation of CrAT, the first due to a metabolic interaction and the latter based on its pharmacology. Singular and combined administration of these two compounds should be evaluated in controlled clinical trials in order to verify our findings in vivo.

The interaction of L-carnitine and PPARα as we could show is of transcriptional importance. Therefore the growth condition where this collaborative interplay is able to trigger the genome deserves deeper investigation. L-carnitine could already be identified as a nutritional modulator of glucocorticoid receptor functions [29]. In our electrophoretic mobility assays we also observed a slight induction at one of the glucocorticoid responsive elements (GRE) at position −421 to −396 of the CrAT promoter, after L-carnitine supplementation, but to a much lesser extend as at the RXRα/PPARα binding site (see Additional file 1: Figure S3). Thus possible interactions of L-carnitine with other nuclear receptors like PPARα and GRE and its molecular basis are worthwhile being investigated in the future.

## Conclusions

Based on our investigations we could present a complete study of the murine CrAT promoter and provide strong evidence for a cooperative interplay of L-carnitine and PPARα for its transcriptional regulation, which undoubtedly is of nutrigenomical importance. Reportergene and electrophoretic mobility assays located a L-carnitine sensitive region within 342 nt upstream the transcription start, that contains one RXRα/PPARα element which is directly responding to L-carnitine supplementation. Super shift assays performed with a polyclonal antibody demonstrated that the muCrAT gene is a PPARα target. These evidences are strengthened by our findings that transcription levels of the PPAR-binding protein (PPARbp) are also inducible by L-carnitine. A direct comparison of L-carnitine with the drug fenofibrate revealed, both are inducers of CrAT transcripts, with one implication that the hyperlipidemic drug fenofibrate exerted a more pronounced effect, based on its pharmacological interaction. Within the investigated CrAT promoter sequence a variety of different putative binding sites for nuclear factors were predicted in silico and verified by experimental approaches. Thus possible interactions of L-carnitine with other nuclear receptors like PPARα and GRE and its molecular basis are worthwhile being investigated in the future. We also could append facts for the bidirectional function of the CrAT promoter in conjunction with the opposing PPP2R4 gene.

## Methods

### Cell culture

The normal embryonic murine liver cell line BNL CL. 2 (ATCC® TIB-73™) was grown at 37°C, 7.5% CO_2_ in DMEM supplemented with 10% fetal calf serum (Sigma Aldrich) and 1% antibiotics (30 mg/l penicillin, 50 mg/l streptomycin sulphate). For the experiments cells were either kept in DMEM containing 10%FCS or dialyzed 10%FCS (dialysis was performed against 1xPBS for 48 h with five buffer changes). L-carnitine (LONZA) and fenofibrate (Lannacher Heilmittel) were added to the culture medium to obtain defined final concentrations as indicated in the experiments.

For reporter gene assays TIB-73 cells grown in 6-well plates were transiently co-transfected with the luciferase reporter constructs mCrAT-1, mCrAT-2 or mCrAT-3 (described in the promoter construct section) as well as with the ß-galactosidase (ß-gal) expressing vector pCMV-ßgal (CLONTECH) for normalization of transfection efficacies.

DNA (0,4 μg pCMV-ßgal and 2 μg of the respective luciferase reporter construct) was mixed with 150 mM NaCl in a total volume of 100 μl for each well. Polyethylenimine (PEI Sigma, 18,5 μl for each well) was mixed with 150 mM NaCl in a total volume of 100 μl for each well. The solution was slowly added to the DNA solution to ensure proper formation of DNA/PEI-complexes, mixed and left at room temperature for 20 min. 200 μl DNA/PEI mix was added to each well and the cells were kept at serum free conditions for ~4 hours.

Afterwards, the cells were washed with DMEM and were allowed to grow in DMEM supplemented with 10% dialyzed FCS and antibiotics for 24 hours. After an additional medium change and cultivation for further 4 hours the cells were treated with various concentrations of L-carnitine (10 μM, 40 μM, 80 μM) and incubated with and without L-carnitine for additional 4 or 24 hours.

### L-carnitine assay

To determine the intracellular L-carnitine levels induced by our cell culture conditions as well as the L-carnitine concentrations in the fetal calf serum (dialyzed/normal) employed in the cell culture the L-carnitine colorimetric/fluorometric assay kit (BioVision) was used. TIB-73 were grown under the conditions described above and 10^6^ cells were homogenized in 100 μl assay buffer exactly as described in the user manual provided by the vendor. In the case of the fetal calf serum 2 μl and 10 μl aliquots were subjected the same assay procedure. Since the enzyme based assay is very sensitive to contaminating protein levels all samples were treated with 4 M HClO_4_ and neutralyzed with 2 M KOH. After centrifugation to remove the precipitated proteins samples were drawn and correlated with an L-carnitine standard curve.

### Quantitative real-time PCR

Total RNA was isolated from cells using Qiagen RNeasy Mini Kit according to the manufacturer’s protocol. RNA concentration and purity were estimated from the optical density at 260 and 280 nm using a NanoDrop spectrophotometer (Thermo Scientific). Three micrograms of RNA were converted to cDNA using RevertAid Reverse Transcriptase (Thermo Scientific) and oligo(dT)_18_ primers. Concentration of primers in each sample was 0.5 μM and 2 μl of a 1:100 dilution of each cDNA was used as template. Parameters for real-time PCR were as follows: 95°C for 10 min, 45 cycles of 95°C for 30 sec, 61°C for 15 sec, 72°C for 40 sec. Amplification of target gene was detected by SYBR Green (Roche LightCycler FastStart DNA Master SYBR Green) and analyzed by ^ΔΔ^-CT method. *β-actin* was used as reference gene. Following primers were used for real-time PCR to quantify mRNA levels: *CrAT* Ps: 5′-GCTCAGCCTCCATAGACTCG-3′, Pas:5′-AGCAATGGCGTAAGAGGTGT-3′; *mu-β-actin* Ps:5′-GCGTGACATCAAAGAGAAG-3′, Pas:5′-AGGAGCCAGAGCAGTAATC-3′;

### Promoter constructs, transfection and reporter gene assays

A genomic DNA-fragment from the complete murine CrAT promoter was generated by PCR amplification. Two primers were designed GP1s 5′ CTCAATGTTCACCCCGCAGC 3′ and GP1as 5′ CAGAGAGGACAGGAGCTCAC 3′ defining a 1433 bp fragment. DNA from a mouse genomic library (3 T6 fibroblasts) served as a template. The purified PCR-generated promoter fragment was ligated into a pBSK(−) vector, subsequently the CrAT promoter DNA fragment in the proper orientation was digested with HindIII and subcloned into the Hind III linearized pGL2-basic reporter gene expression vector (Promega), the resulting plasmid clones were analyzed by restriction digestion and sequence analysis. For transfection into mammalian host cells two constructs were established: The construct pGL2-mCrAT-1 was generated with primers GP1s 5′-CTCAATGTTCACCCCGCAGC-3′ and mCrAT-1as 5′-GGTTCTAGGTTCAAGGTCGC-3′ included the promoter fragment (−763/+15). The second construct pGL2-mCrAT-2, generated with primers mCrAT2s 5′-GAGTGACGTTCAAGGACACC −3′ and mCrAT1as 5′-GGTTCTAGGTTCAAGGTCGC −3′ carried the promoter segment (−342/+15). The third construct pGmCrAT-3, generated with primers GP1s 5′ -CTCAATGTTCACCCCGCAGC-3′ mCrAT3as 5′-GTCCTTGAACGTCACTCTAGG −3′contained the promoter segment (−778/-328).

After the given time points (described above) the cells were harvested, transferred to a microfuge tube, followed by two rounds of freeze – thaw circles with liquid nitrogen versus 37°C to ensure complete lysis. The cell lysate was centrifuged at 15.000 rpm, 4°C for 3 min and the supernatant was transferred to a fresh microfuge tube. 200 μl cell extract was mixed with 16 μl luciferin (Applichem), 4 μl ATP (0,1 M) and 1 μl DTT (1 M), vortexed and the luciferase activity in each lysate was measured by a Berthold LB953 luminometer.

### In silico analysis of mouse CrAT promoter and 5′UTR for putative nuclear factor binding sites

To identify putative binding sites of transcriptional active nuclear factors in the mouse *CrAT* promoter 850 kb of the 5′ flanking region of mouse *CrAT* from −700 to +150 relative to the transcription start were analysed using the online tools TESS (http://www.cbil.upenn.edu/cgi-bin/tess/tess) and PATCH (http://www.gene-regulation.com/cgi-bin/pub/programs/patch/bin/patch.cgi).

### Electrophoretic mobility shift assay (EMSA)

Nuclear extracts were prepared from 2x10^6^ TIB-73 cells after treatment with or without dialyzed FCS and in presence or absence of L-carnitine (40–120 μM) or fenofibrate (10–40 μM) after an established protocol [30]. Proteinase inhibitors (Complete Proteinase Inhibitor Mix, Roche) were added according the manufacturer’s protocol. Complementary synthetic oligonucleotides corresponding to the RXR binding site in the CrAT promoter were obtained from VBC Biotech (Vienna, Austria) (fwd 5′-AGCGCCTACCGTTGTGACCCCG-3; rev 5′-CGGGGTCACAACGGTAGGCGCT-3′) Double stranded oligonucleotides were labeled with γ-^32^P-ATP by T4 polynucleotide kinase (PNK) reaction. The protein-DNA binding mixtures contained labelled probe, nuclear extracts, sonicated salmon sperm DNA as unspecific competitor, 4% glycerol, 20 mM TRIS pH 8, 60 mM KCl, 5 mM MgCl_2_, 500 μg/ml BSA. Binding reactions were incubated for 30′ to 1 h and then resolved in 5% non-denaturating acrylamide gels in 1x TBE buffer. Electrophoresis was carried out at 120 V for 360 min at 4°C. For supershift analysis antibody solution (anti-PPARα, sc-9000, Santa Cruz Biotechnology) was added after 15′ of preincubation of nuclear extracts with oligonucleotides.

### Western blot analysis

PPARα protein levels from nuclear extracts were analysed by Western blot. Nuclear extracts were prepared after an established protocol as described above. Aliquots containing 25 μg of nuclear extract were loaded on to a 10% SDS polyacrylamid gel. After electrophoresis proteins were transferred to a nitrocellulose membrane. The membrane was probed with antibodies against PPARα (sc-9000, Santa Cruz Biotechnology) and GAPDH (sc-47724, Santa Cruz Biotechnology). In the case of the CrAT western blot 10 μg whole cell protein extracts were separated on 12% SDS polyacrylamid gels and probed with CrAT antibody (Abcam ab91478) and GAPDH (sc-47724, Santa Cruz Biotechnology). The membranes were then processed with HRP conjugated secondary antibodies specific for the appropriate species. Proteins were visualized with Western Lightning ECL kit (Perkin Elmer). The statistical analyses were carried by SPSS software (IBM) using the *t*-test subroutine for independent samples (student’s *T* test) to calculate the p-values given in the figure legends. In the case of Figure 4 we performed a Kruskal-Wallis test with GraphPad Prism (GraphPad Software Inc.).

### Southwestern analysis

For southwestern analysis nuclear extracts and oligonucleotides were prepared as described above. Protein samples were separated by SDS-PAGE and then transferred to nitrocellulose membrane at 110 V for 90 min at 4°C. Blotted proteins were renatured in 1xTNED buffer (10 mM TRIS pH 7.5, 50 mM NaCl, 0,1 mM EDTA, 1 mM DTT) with 5% skim milk for 24 h. The membrane was incubated with γ-^32^P-ATP marked oligonucleotides in 1xTNED with 2.5% skim milk for another 24 h. Blot was exposed to chemiluminescent sensitive films visualized via autoradiography. Protein size was determined and further on analyzed via *molwsearch* using *Transfac* database (http://www.gene-regulation.com/cgi-bin/pub/programs/molwsearch/molwsearch.cgi).

## Electronic supplementary material

Additional file 1: Figure S1: qPCR of human PPP2R4 from the human liver cell line HepG2: (A) cells were treated 24 h with dialyzed FCS and supplemented afterwards for 4 hours with L-carnitine (80 μM). Values show mean SD, n = 4, ***p < 0.001 vs. DMEM + 10% FCS. (B) Cells were grown in DMEM + 10%FCS for 24 hours and afterwards treated with fenofibrate (10–40 μM) for four hours. Values represent means ± SD (n = 4). Supplemented cultures were compared to physiological control (DMEM + 10% FCS) ***p < 0.001. Methods: Human liver cell line HepG2 was treated as described in the methods section for TIB-73 cells. For quantitative PCR given protocols as described in the methods sections were followed. Following primers were used: PPP2R4 Ps: 5′CAAGAGTGAAAGGCGAGACG3′, Pas:5′CCATGTCTGGAACTGTGTGG′; ß-actin Ps: 5′GATGAGTATGCCTGCCGTGTG3′, Pas: 5′TCAACTGGTCTCAA-GTCAGTG3′. **Figure S2.** Electrophoretic mobility super shift assay with anti-RXRα and anti-PPARγ: Nuclear extracts from TIB-73 cells supplemented with increasing concentrations of L-carnitine were incubated with -32P-labeled oligonucleotides representing the RXRα-binding site with anti-RXRα and anti-PPAR as indicated. No mitigation effect was observable as seen with anti-PPARα antibodies in Figure 5. **Figure S3.** Electrophoretic mobility shift assay of one of the CrAT promoter GR-binding sites: Nuclear extracts from TIB-73 cells supplemented with increasing concentrations of L-carnitine were incubated with a -32P-labeled oligonucleotide representing the GR-binding site sense: 5′ GTCAACAGTTGTGTTCTCCTGCCATTC3′. (PDF 118 KB)

## References

[1] Vaz FM, Wanders RJA (2002). Carnitine biosynthesis in mammals. Biochem J.

[2] Strijbis K, Vaz F, Distel B (2010). Enzymology of the carnitine biosynthesis pathway. IUBMB Life.

[3] Tanphaichitr V, Broquist HP (1973). Role of lysine and N-trimethyllysine in carnitine biosynthesis. J Biol Chem.

[4] Ramsay RR, Gandour RD, van der Leij FR (2001). Molecular enzymology of carnitine transfer and transport. Biochim Biophys Acta Protein Struct Mol Enzymol.

[5] Kerner J, Hoppel C (2000). Fatty acid import into mitochondria. Biochem Biophys Acta Mol Cell Biol Lipids.

[6] Mingrone G (2004). Carnitine in type 2 diabetes. Ann N Y Acad Sci.

[7] DeFronzo RA (1999). Pharmacologic therapy for type 2 diabetes mellitus. Ann Intern Med.

[8] Wu P, Inskeep K, Bowker-Kinley MM, Popov KM, Harris RA (1999). Mechanism responsible for inactivation of skeletal muscle pyruvate dehydrogenase complex in starvation and diabetes. Diabetes.

[9] Brunner S, Kramar K, Denhardt DT, Hofbauer R (1997). Cloning and characterization of murine carnitine acetyltransferase: evidence for a requrement during cell cycle progression. Biochem J.

[10] van der Leij FR, Huijkman NCA, Boomsma C, Kuipers JRG, Bartelds B (2000). Genomics of the human carnitine acyltransferase genes. Mol Genet Metab.

[11] Zammit VA, Ramsay RR, Bonomini M, Arduini A (2009). Carnitine, mitochondrial function and therapy. Adv Drug Deliv Rev.

[12] Noland RC, Koves TR, Seiler SE, Lum H, Lust RM, Ilkayeva O, Stevens RD, Hegardt FG, Muoio DM (2009). Carnitine insufficiency caused by aging and overnutrition compromises mitochondrial performance and metabolic control. J Biol Chem.

[13] Power RA, Hulver MW, Zhang JY, Dubois J, Marchand RM, Ilkayeva O, Muoio DM, Mynatt RL (2007). Carnitine revisited: potential use as adjunctive treatment in diabetes. Diabetologia.

[14] Muoio DM, Noland RC, Kovalik J-P, Seiler SE, Davies MN, DeBalsi KL, Ilkayeva OR, Stevens RD, Kheterpal I, Zhang J, Covington JD, Bajpeyi S, Ravussin E, Kraus W, Koves TR, Mynatt RL (2012). Muscle-Specific Deletion of Carnitine Acetyltransferase Compromises Glucose Tolerance and Metabolic Flexibility. Cell Metabolism.

[15] Godarova A, Litzlbauer E, Brunner S, Agu A, Lohninger A, Hofbauer R (2005). L-Carnitine regulates mRNA expression levels of the carnitine acyltransferases - CPT1A, CPT2 and CRAT. Chem Mon.

[16] Rakhshandehroo M, Sanderson LM, Matilainen M, Stienstra R, Carlberg C, de Groot PJ, Müller M, Kersten S (2007). Comprehensive Analysis of PPARalpha-Dependent Regulation of Hepatic Lipid Metabolism by Expression Profiling. PPAR Research.

[17] Tachibana K, Kobayashi Y, Tanaka T, Tagami M, Sugiyama A, Katayama T, Ueda C, Yamasaki D, Ishimoto K, Sumitomo M, Uchiyama Y, Kohro T, Sakai J, Hamakubo T, Kodama T, Doi T (2005). Gene expression profiling of potential peroxisome proliferator-activated receptor (PPAR) target genes in human hepatoblastoma cell lines inducibly expressing different PPAR isoforms. Nuclear Receptor.

[18] Janssens V, Van Hoof C, De Baere I, Merlevede W, Goris J (1999). Functional analysis of the promoter region of the human phosphotyrosine phosphatase activator gene: Yin Yang 1 is essential for core promoter activity. Biochem J.

[19] Janssens V, Van Hoof C, De Baere I, Merlevede W, Goris J (2000). The phosphotyrosyl phosphatase activator gene is a novel p53 target gene. J Biol Chem.

[20] Hofer-Litzlbauer E: *Biochemical and genetical consequences of carnitine deficiency caused by downregulation of the carnitine acyltransferase genes.**Doctoral Thesis.* Vienna: Medical University of Vienna; 2005.

[21] Cook GA, Edwards TL, Jansen MS, Bahouth SW, Wilcox HG, Park EA (2001). Differential Regulation of Carnitine Palmitoyltransferase-I Gene Isoforms (CPT-I alpha and CPT-Ibeta) in the Rat Heart. J Mol Cell Cardiol.

[22] Barrero MJ, Camarero N, Marrero PF, Haro D (2003). Control of human carnitine palmitoyltransferase II gene transcription by peroxisome proliferator-activated receptor through a partially conserved peroxisome proliferator-responsive element. Biochem J.

[23] Dayoub R, Groitl P, Dobner T, Bosserhoff AK, Schlitt H-J, Weiss TS (2010). Foxa2 (HNF-3[beta]) regulates expression of hepatotrophic factor ALR in liver cells. Biochem Biophys Res Commun.

[24] Li JL, Wang QY, Luan HY, Kang ZC, Wang CB (2012). Effects of L-carnitine against oxidative stress in human hepatocytes: involvement of peroxisome proliferator-activated receptor alpha. J Biomed Sci.

[25] Gerondaes P, Alberti GMM, Loranne A (1988). Fatty acid metabolism in hepatocytes cultured with hypolipidaemic drugs. Biochem J.

[26] Ide T, Shimano H, Yoshikawa T, Yahagi N, Amemiya-Kudo M, Matsuzaka T, Nakakuki M, Yatoh S, Iizuka Y, Tomita S, Ohashi K, Takahashi A, Sone H, Gotoda T, Osuga J, Ishibashi S, Yamada N (2003). Cross-Talk between Peroxisome Proliferator-Activated Receptor (PPAR) alpha and Liver X Receptor (LXR) in Nutritional Regulation of Fatty Acid Metabolism. II. LXRs Suppress Lipid Degradation Gene Promoters through Inhibition of PPAR Signaling. Mol Endocrinol.

[27] Roche T, Hiromasa Y (2007). Pyruvate dehydrogenase kinase regulatory mechanisms and inhibition in treating diabetes, heart ischemia, and cancer. Cell Mol Life Sci.

[28] Consoli A (1992). Role of liver in pathophysiology of NIDDM. Diabetes Care.

[29] Alesci S, De Martino MU, Mirani M, Benvenga S, Trimachi F, Kino T, Chrousos GP (2003). L-Carnitine: a nutritional modulator of glucocorticoid receptor functions. FASEB J.

[30] Siu FKY, Lee LTO, Chow BKC (2008). Southwestern blotting in investigating transcriptional regulation. Nat Protoc.

