# Supplementary material for: L-carnitine and PPARα-agonist fenofibrate are involved in the regulation of Carnitine Acetyltransferase (CrAT) mRNA levels in murine liver cells
Source: BMC Genomics. 2014 Jun 24;15(1):514. doi: 10.1186/1471-2164-15-514 (PMC4089027; doi:10.1186/1471-2164-15-514)
Supplement: Supplementary file 1 — Additional file 1: Figure S1: qPCR of human PPP2R4 from the human liver cell line HepG2: (A) cells were treated 24 h with dialyzed FCS and supplemented afterwards for 4 hours with L-carnitine (80 μM). Values show mean SD, n = 4, ***p < 0.001 vs. DMEM + 10% FCS. (B) Cells were grown in DMEM + 10%FCS for 24 hours and afterwards treated with fenofibrate (10–40 μM) for four hours. Values represent means ± SD (n = 4). Supplemented cultures were compared to physiological control (DMEM + 10% FCS) ***p < 0.001. Methods: Human liver cell line HepG2 was treated as described in the methods section for TIB-73 cells. For quantitative PCR given protocols as described in the methods sections were followed. Following primers were used: PPP2R4 Ps: 5′CAAGAGTGAAAGGCGAGACG3′, Pas:5′CCATGTCTGGAACTGTGTGG′; ß-actin Ps: 5′GATGAGTATGCCTGCCGTGTG3′, Pas: 5′TCAACTGGTCTCAA-GTCAGTG3′. Figure S2. Electrophoretic mobility super shift assay with anti-RXRα and anti-PPARγ: Nuclear extracts from TIB-73 cells supplemented with increasing concentrations of L-carnitine were incubated with -32P-labeled oligonucleotides representing the RXRα-binding site with anti-RXRα and anti-PPAR as indicated. No mitigation effect was observable as seen with anti-PPARα antibodies in Figure 5. Figure S3. Electrophoretic mobility shift assay of one of the CrAT promoter GR-binding sites: Nuclear extracts from TIB-73 cells supplemented with increasing concentrations of L-carnitine were incubated with a -32P-labeled oligonucleotide representing the GR-binding site sense: 5′ GTCAACAGTTGTGTTCTCCTGCCATTC3′. (PDF 118 KB) [file 12864_2014_6206_MOESM1_ESM.pdf]

Supplementary figures:

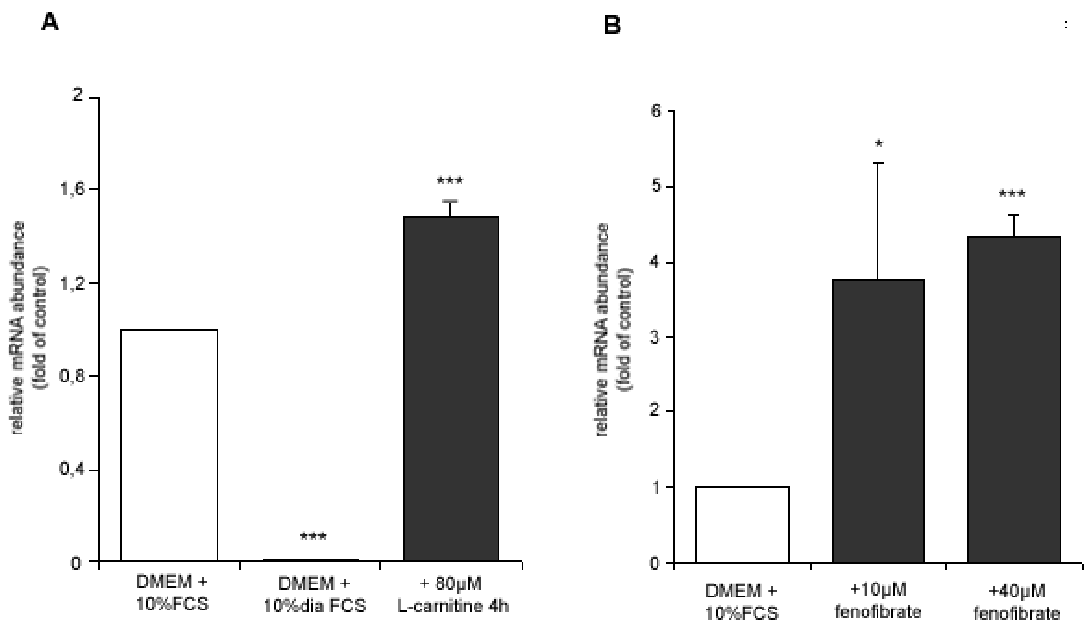

Supplementary Figure 1: qPCR of human PPP2R4 from the human liver cell line HepG2. (A) cells were treated 24h with dialyzed FCS and supplemented afterwards for 4 hours with L-carnitine (80 µM). Values show mean ± SD, n=4, ***p<0.001 vs. DMEM+10%FCS. **(B)** Cells were grown in DMEM+10%FCS for 24 hours and afterwards treated with fenofibrate (10-40 µM) for four hours. Values represent means ± SD (n=4). Supplemented cultures were compared to physiological control (DMEM+10%FCS) *** p<0.001.

Methods: Human liver cell line HepG2 was treated as described in the methods section for TIB-73 cells. For quantitative PCR given protocols as described in the methods sections were followed. Following primers were used: PPP2R4 Ps: 5'CAAGAGTGAAAGGCGAGACG3', Pas:5'CCATGTCTGGAACTGTGTGG'; ß-actin Ps: 5'GATGAGTATGCCTGCCGTGTG3', Pas: 5'TCAACTGGTCTCAA-GTCAGTG3'.

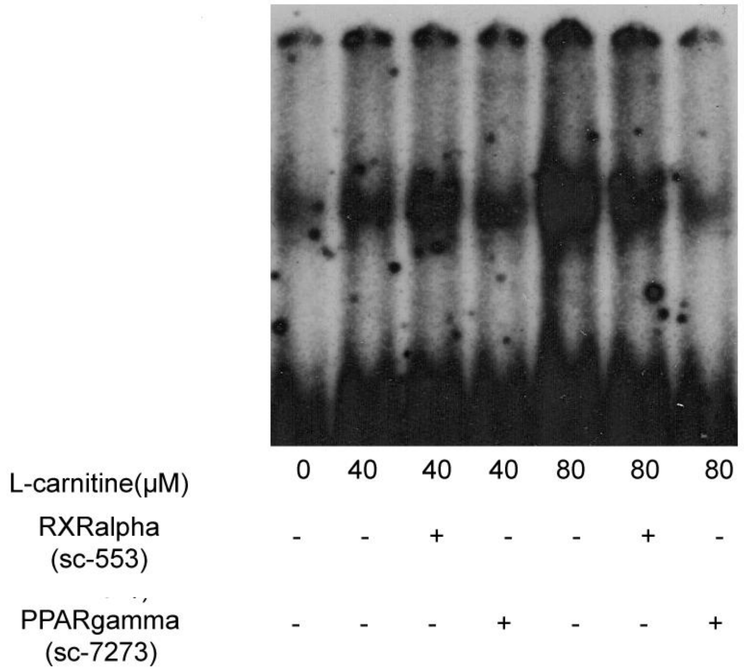

Supplementary Figure 2: EMSA. Nuclear extracts from TIB-73 cells supplemented with increasing concentrations of L-carnitine were incubated with γ-^{32}P-labeled oligonucleotides representing the RXRα-binding site with anti-RXRα and anti-PPARγ as indicated. No mitigation effect was observable as seen with anti-PPARα antibodies in figure 5.

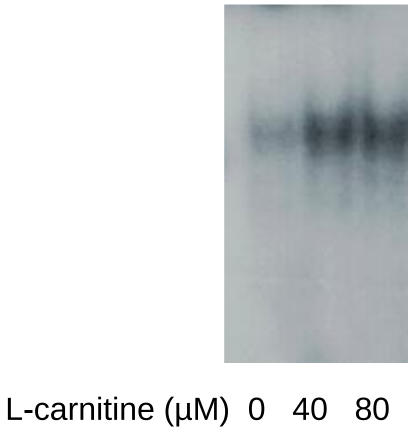

Supplementary Figure 3: Nuclear extracts from TIB-73 cells supplemented with increasing concentrations of L-carnitine were incubated with a γ-^{32}P-labeled oligonucleotide representing the GR-binding site sense: 5´ GTCAACAGTT-GTGTTCTCCTGCCATTC 3’
